# Analysis of vasoactive and oxidative stress indicators for evaluating the efficacy of continuous positive airway pressure, and relation of vasoactive and oxidative stress indicators and cardiac function in obstructive sleep Apnea Syndrome patients

**DOI:** 10.5937/jomb0-42944

**Published:** 2024-04-23

**Authors:** Xiaohong Ni, Jinhua Wang, Yu Tian, Hongyan Ke, Yuangao Liao, Yanwen Lv

**Affiliations:** 1 Huanggang Central Hospital, Department of Neurology, Huanggang City, China

**Keywords:** obstructive sleep apnea syndrome, continuous positive airway pressure, vasoactive factors, oxidative stress indicators, therapeutic efficacy, cardiac function, sindrom opstruktivne sleep apneje, kontinuirani pozitivni pritisak na disajne puteve, vazoreaktivni faktori, pokazatelji oksidativnog stresa, terapijska efikasnost, srčana funkcija

## Abstract

**Background:**

Obstructive Sleep Apnea Syndrome (OSAS) is a breathing disorder during sleep. The work was to evaluate the relationship between vasoactive and oxidative stress indicators and cardiac function in Obstructive Sleep Apnea Syndrome (OSAS) patients.

**Methods:**

OSAS patients (n=120) were treated with CPAP from May 2021 to June 2022. According to the clinical efficacy, the patients were divided into effective and ineffective groups. Vasoactive factors and oxidative stress indices were compared between the two groups to evaluate their clinical efficacy. The changes in cardiac function indices in the two groups were tested, and the correlation between vasoactive factors and oxidative stress indices and cardiac function was analysed.

**Results:**

The effective rate of CPAP was 63.33% (76/120). Ang II, ET-1, and MDA levels were lower, and the SOD level was higher in the effective group than in the ineffective group after treatment. The AUC of the four indicators was all greater than 0.75. LPWT and IVST values of the effective group were lower than the ineffective group. A positive correlation was identified between the levels of Ang II, ET-1, and MDA with LPWT, between levels of ET-1 and MDA with IVST, and a negative correlation between SOD with LPWT and IVST.

**Conclusions:**

CPAP treatment can effectively improve vascular activity and reduce the oxidative stress response in OSAS patients, and the combined detection of vasoactive factors and oxidative stress indicators is valuable for evaluating the efficacy of CPAP and is related to the cardiac function of patients.

## Abstract (sr)

**Uvod:**

Sindrom opstruktivne sleep apneje (OSAS) je poremećaj disanja tokom spavanja. Cilj ovog istraživanja bio je da se proceni odnos između vazoreaktivnih i pokazatelja oksidativnog stresa i srčane funkcije kod pacijenata sa sindromom opstruktivne sleep apneje (OSAS).

**Metode:**

Pacijenti sa OSAS-om (n=120) su tretirani kontinuiranim pozitivnim pritiskom na disajne puteve (CPAP) od maja 2021. do juna 2022. godine. Na osnovu kliničke efikasnosti, pacijenti su podeljeni u efikasnu i neefikasnu grupu. Vazoreaktivni faktori i pokazatelji oksidativnog stresa su upoređeni između ove dve grupe kako bi se procenila njihova klinička efikasnost. Testirane su promene u pokazateljima srčane funkcije kod obe grupe, a analizirana je i veza između vazoreaktivnih faktora, pokazatelja oksidativnog stresa i srčane funkcije.

**Rezultati:**

Efikasnost CPAP-a je bila 63,33% (76/120). Nivoi Ang II, ET-1 i MDA su bili niži, dok je nivo SOD bio viši u efikasnoj grupi u odnosu na neefikasnu grupu nakon tretmana. AUC svih četiri pokazatelja bio je veći od 0,75. Vrednosti LPWT i IVST kod efikasne grupe su bile niže u poređenju sa neefikasnom grupom. Uočena je pozitivna korelacija između nivoa Ang II, ET-1 i MDA sa LPWT između nivoa ET-1 i MDA sa IVST, i negativna korelacija između SOD sa LPWT i IVST.

**Zaključak:**

CPAP tretman može efikasno poboljšati vaskularnu aktivnost i smanjiti odgovor na oksidativni stres kod pacijenata sa OSAS, a kombinovana detekcija vazoaktivnih faktora i indikatora oksidativnog stresa je dragocena za procenu efikasnosti CPAP-a i povezana je sa srčanom funkcijom pacijenata.

## Introduction

Obstructive Sleep Apnea Syndrome (OSAS) is a breathing disorder that occurs during sleep, which is relatively common and easily overlooked [1]. At present, it is believed that OSAS may cause irreversible damage to the function of multiple systems of the whole body and significantly impact the cardiac function of patients. Relevant studies have pointed out that OSAS patients often have abnormal oxidative stress responses [2] [3]. Repeated nocturnal hypoxia in patients with OSAS can lead to excessive oxidative stress in the body, especially in the lungs, which can induce airway hyper-responsiveness and bronchial asthma, thereby aggravating the disease. It has been reported that in the pathological process of OSAS, hypoxemia destroys cardiomyocytes through oxidative stress, abnormal ion channels, activation of inflammatory factors, and release of vasoactive factors, which affects the myocardial function of patients, and then leads to cardiac dysfunction in patients [4] [5]. Continuous positive airway pressure (CPAP) is one of the primary methods for treating OSAS. It can improve the symptoms of apnea and hypoxia in patients by keeping the upper airway open [6] [7]. However, there is no report on the effect of this method on vascular function and oxidative stress in patients with OSAS. Therefore, this study guided us to analyse the relationship between vasoactive/oxidative stress and therapeutic efficacy and cardiac function in patients with OSAS after CPAP and to provide a reference for the clinical treatment of the disease.

## Materials and methods

### Clinical data

OSAS patients, in 120 cases, were recruited as the research subjects from May 2021 to June 2022.

*Inclusion criteria*: meeting the diagnostic criteria of OSAS [7]; receiving CPAP treatment; no previous history of hypertension drug treatment; complete clinical data.

*Exclusion criteria*: central sleep apnea syndrome; previous CPAP treatment and palatopharyngoplasty surgery; Severe hypertension (N3 stage); renal failure; Left ventricle ejection fraction <60%; heart diseases such as severe valvular regurgitation and moderate to severe valvular stenosis; smoking >10 d.

### CPAP treatment and evaluation of clinical efficacy

Autoset DS500 (Philips Respironics) and ResMed S10, as home intelligent CPAP (AutoCPAP) treatment machines to monitor patients and improve the patient's respiratory condition. According to the evaluation criteria of the Chinese Medical Association Otolaryngology Branch [8], markedly effective: apnea-hypopnea index (AHI) 20 or AHI reduction ≥50%, and the symptoms were significantly improved; Effective: AHI reduction ≥25%, symptoms were improved; Ineffective: AHI reduction <25%, symptoms did not improve. Total effective rate=effective rate+efficiency.

### Serum index detection and cardiac ultrasonography

Measurement of angiotensin II (Ang II) and endo thelin-1 (ET-1) was by enzyme-linked immunosorbent assay (ELISA), that of malondialdehyde (MDA) and superoxide dismutase (SOD) was by thiobarbituric acid method and xanthine oxidase method, respectively.

US GE-VIVD3 ultrasonic diagnostic apparatus was used to perform transthoracic two-dimensional M-mode and colour Doppler echocardiography. The patient was placed in the left lateral position, and the long-axis left ventricular section of the sternum was taken to observe the heart structure, left ventricular end-diastolic dimension (LVDD), left ventricular endsystolic dimension (LVSD), left posterior wall thickness (LPWT) and interventricular septal thickness (IVST).

Vasoactive factors and oxidative stress indicators between the effective group and the ineffective group were compared, and their value for evaluating clinical efficacy was analysed. The changes in cardiac function indices in the effective and ineffective groups were compared, and the correlation between vasoactive factors and oxidative stress indices and cardiac function was analysed. 

### Statistical method

Data analysis was carried out by SPSS 22.0 software. Enumeration data were expressed in % and compared by χ^2^ test. Measurement data were reported by mean±standard deviation after the normality test and compared by student t-test. Graph drawing was by GraphPad Prism 5, clinical efficacy evaluation value analysis was by ROC curve, and correlation analysis was by Spearman test. *P*<0.05 meant the difference was statistically significant.

## Results

### General data in the effective group and the ineffective group

The effective rate of CPAP in the treatment of OSAS was 63.33% (76/120). The two groups had No significant difference in general data (*P*>0.05, Table 1).

**Table 1:** General data in the effective group and the ineffective group. Data were shown in n (%) or Mean±SD deviation after the normality test and compared by χ^2^ or student t-test.

Groups	Effective<br>(n=76)	Ineffective<br>(n=44)	χ^2^/t	P
Gender			0.038	0.845
Male	47 (61.8)	28 (63.6)		
Female	29 (38.2)	16 (36.3)		
Age (year)	54.19±6.31	55.04±6.47	0.705	0.482
Course of disease (month)	3.26±0.41	3.18±0.45	0.994	0.322
BMI (kg/m^2^)	22.93±2.06	22.71±2.73	0.499	0.619
Smoking	26 (34.2)	17 (38.6)	0.237	0.626
Drinking	21 (27.6)	14 (31.8)	0.236	0.627
Combined hypertension	15 (19.7)	7 (15.9)	0.273	0.602
Combined hyperlipidemia	13 (17.1)	8 (18.2)	0.022	0.881
Combined diabetes	9 (11.8)	5 (11.3)	0.006	0.937
FEV1 (%)	86.31±3.28	85.47±2.96	1.4	0.164
FVC (%)	86.05±2.72	85.29±2.53	1.513	0.133
Neck circumference (cm)	42.95±1.79	43.1±1.41	0.477	0.635
Waistline (cm)	95.27±8.15	96.46±7.94	0.778	0.438

### Vasoactive factors between the effective group and the ineffective group before and after CPAP treatment 

**Figure 1:**
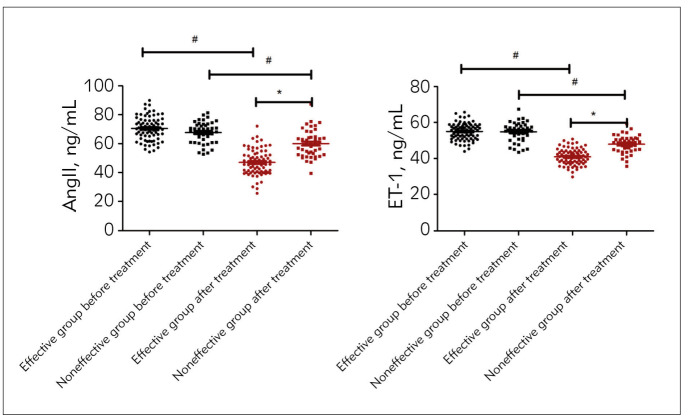
General data in the effective group and the ineffective group<br>Data were shown by Mean±SD, Comparison with the same group before and after treatment, # P<0.05 Comparison between groupsafter treatment, * *P*<0.05.

Levels of Ang II and ET-1 exhibited no distinct difference between the effective group and the ineffective group before treatment (*P* > 0.05); lower levels of Ang II and ET-1 were found in the two groups after treatment than before treatment, and Ang II and ET-1 levels were lower in the effective group than the ineffective group (*P*<0.05, Figure 1).

### Changes of oxidative stress indices in the effective group and the ineffective group before and after treatment

**Figure 2:**
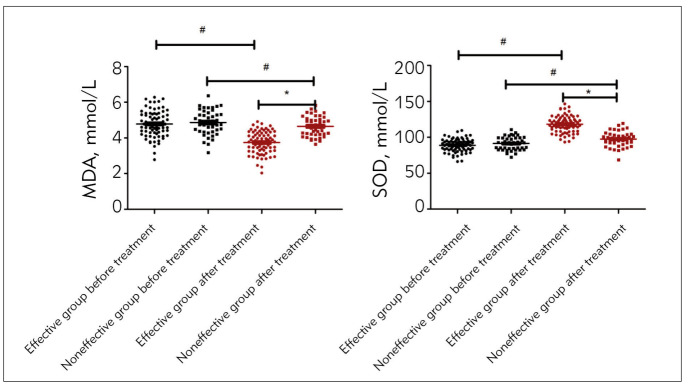
Changes of oxidative stress indices before and after treatment in effective group and ineffective group<br>Data were shown by Mean ± SD, Comparison with the same group before and after treatment, # *P*<0.05<br>Comparison between groups after treatment, * *P* < 0.05.

Neither MDA nor SOD levels were significantly different in the effective group and the ineffective group (*P* > 0.05); after treatment, the change of MDA level in the effective group was lower than that in the ineffective group, while that of SOD level was in an opposite situation (*P* <0.05, Figure 2).

### Analysis of the evaluation value of vasoactive factors and oxidative stress indicators on the efficacy of CPAP treatment 

**Figure 3:**
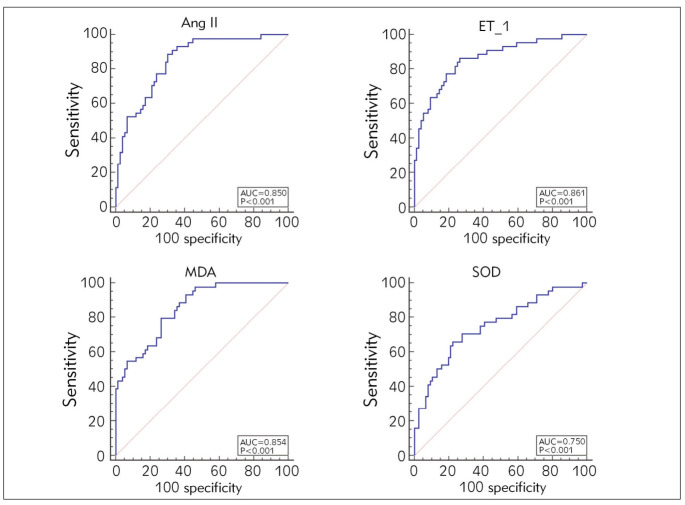
Analysis of the evaluation value of vasoactive factors and oxidative stress indicators on the efficacy of CPAP treatment.

AUC of Ang II, ET-1, MDA, and SOD levels were all greater than 0.75 (Figure 3).

### Comparison of cardiac function indices between the effective group and the ineffective group after treatment

**Figure 4:**
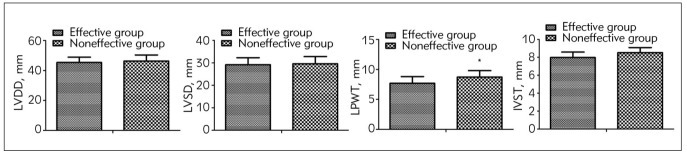
Comparison of cardiac function indices between the effective and ineffective groups after treatment.<br>Comparison with the effective group, * *P*<0.05

No difference was observed in LVDD and LVSD between the two groups after treatment (*P*>0.05); LPWT and IVST values in the effective group after treatment were lower than those in the ineffective group (*P*<0.05, Figure 4).

### Correlation analysis of vasoactive factors, oxidative stress indices, and cardiac function indices in OSAS patients

**Figure 5:**
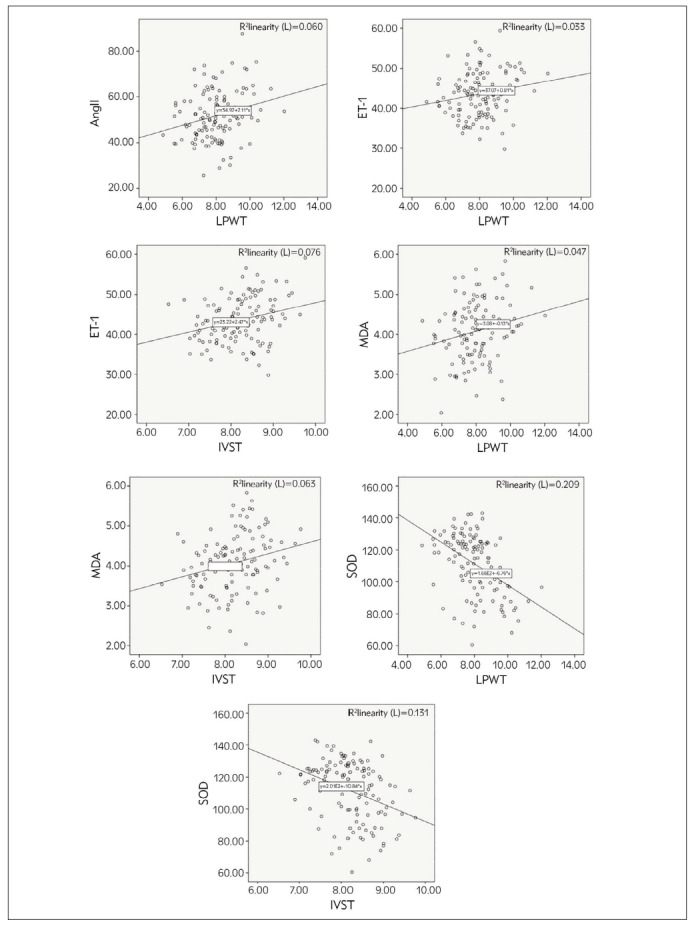
Correlation analysis of vasoactive factors, oxidative stress indices, and cardiac function indices in OSAS patients.

A positive correlation was identified between the levels of Ang II, ET-1, and MDA with LPWT, between levels of ET-1 and MDA with IVST, and a negative correlation between SOD with LPWT and IVST (*P* < 0.05, Table 2, Figure 5).

**Table 2:** Correlation analysis of vasoactive factors, oxidative stress indices and cardiac function indices in OSAS patients. Correlation analysis was by the Spearman test.

Indicators	LVDD		LVSD		LPWT		IVST	
	*r*	*P*	*r*	*P*	*r*	*P*	*r*	*P*
Ang II	-0.012	0.897	0.103	0.264	0.246	0.007	0.155	0.091
ET-1	0.105	0.254	0.049	0.597	0.183	0.046	0.276	0.002
MDA	0.096	0.296	0.091	0.322	0.217	0.017	0.25	0.006
SOD	-0.014	0.875	-0.066	0.473	-0.457	0	-0.363	

## Discussion

OSAS is characterised by snoring, repeated apnea, and hypoxia during sleep, which can cause cardiovascular and cerebrovascular diseases such as coronary heart disease [9]. Studies have found that the antioxidant capacity of OSAS patients is reduced. OSAS patients are often accompanied by hypoxia due to respiratory disorders. The reperfusion of oxygen after hypoxia significantly increases the oxygen consumption of activated neutrophils and generates many oxygen free radicals, thereby causing cell damage [10] [11]. Studies have also shown that OSAS is related to oxidative stress [12], and patients with OSAS have abnormal secretion of vasoactive factors [13]. In the pathological process of OSAS, hypoxemia damages vascular endothelial cells through oxidative stress and the release of vasoactive factors, resulting in tissue ischemia and hypoxia, which leads to cardiac systolic and diastolic dysfunction and arrhythmia in patients. It has been reported that long-term sleep hypoxemia in OSAS patients can lead to secondary polycythemia, and the interaction of various reasons can increase blood viscosity, eventually leading to damage to the vascular endothelium, which increases the incidence of thromboembolic diseases [14] [15]. CPAP, a certain gas pressure, is used to keep the patient's upper airway open during sleep, thereby improving the hypoxia state of the body [16]. This study found that the effective rate of CPAP in the treatment of OSAS was 63.33%, which was not much different from related reports, indicating that CPAP can improve the clinical symptoms of OSAS patients to a certain extent. Also, our study demonstrated that after treatment, the levels of Ang II, ET-1, and MDA in the effective group were lower than those in the ineffective group. The SOD level was higher than that in the ineffective group, indicating that CPAP treatment can improve the vascular function of patients with OSAS and reduce the body's oxidative stress response. A relevant report has stated that after effective treatment of OSAS patients, the hypoxia condition can be improved, the release of oxygen free radicals by neutrophils can be reduced, and the body's antioxidant capacity can be increased simultaneously. Therefore, the therapeutic effect may be evaluated by detecting the changes in the oxidative stress state of patients [17]. In this study, it was found that the AUCs of Ang II, ET-1, MDA, and SOD levels were all greater than 0.75, indicating that Ang II, ET-1, MDA, and SOD levels were all valuable in evaluating the efficacy of CPAP treatment.

OSAS is apnea and hypoventilation caused by upper airway collapse and obstruction, accompanied by symptoms such as daytime sleepiness and fatigue [18]. OSAS patients suffer from a variety of cardiovascular diseases, such as hypertension, arrhythmia, and myocardial ischemia, due to factors such as increased upper airway resistance during sleep breathing, repeated hypoxemia and hypercapnia, abnormal sympathetic nerve activation, heart failure, and independent risk factors for sudden cardiac death [19] [10]. OSAS patients are very common, but abnormal changes in the cardiovascular system in OSAS patients have the characteristics of insidious development. If OSAS patients are not treated in time, the damage to their cardiovascular system will be irreversible [20] [21]. Therefore, early detection, treatment, and treatment adherence have important clinical significance for protecting the function of the cardiovascular system in patients with OSAS. Studies have confirmed that CPAP treatment can reduce the risk of cardiovascular disease in patients with OSAS, delay the progression of complicated cardiovascular disease, and improve prognosis [22]. This study found that LPWT and IVST values in the effective group were lower than those in the ineffective group after treatment, indicating that CPAP therapy can improve cardiac function in patients with OSAS. The reason is that CPAP treatment can improve the regulation of the central nervous system on respiratory function, correcting repeated hypoxemia during sleep, relieving vasospasm, reducing the patient's cardiac load and improving cardiac function.

The occurrence of respiratory disorders in patients with OSAS can initiate the oxidative stress response of the body. Reactive oxygen radicals have been associated with many diseases, including autoimmune diseases like rheumatoid arthritis, diabetes mellitus, atherosclerosis, obesity, hypertension and cardiovascular diseases such as ischemia [23] [24]. The activation of redox-sensitive genes can promote the production of various cell adhesion molecules, which can cause myocardial ischemia, suggesting that the oxidative stress state of patients may be related to cardiac function. The present study indicated that the levels of Ang II, ET-1, and MDA in OSAS patients were positively correlated with LPWT values, the levels of ET-1 and MDA were positively correlated with IVST values, and SOD was negatively correlated with LPWT and IVST values, indicating that vasoactive factors and oxidative stress indicators are related to the cardiac function of patients, which is consistent with related research [25]. This is mainly because oxidative stress can promote local inflammation through oxidation, induce vascular endothelial damage, reduce vascular function, and ultimately participate in heart disease by changing the expression of vascular-related factors and affecting signal transduction pathways.

### Conclusion and future directions

CPAP treatment can effectively improve vascular activity and reduce the oxidative stress response in OSAS patients, and the combined detection of vasoactive factors and oxidative stress indicators is valuable for evaluating the efficacy of CPAP and is related to the cardiac function of patients. Therefore, further studies in surgical patients are required for indications of antioxidative therapy in the prevention and diagnostics of Obstructive Sleep Apnea Syndrome. According to the available measured data, surgical patients are closely associated with changes in the redox status and minimising oxidative stress is necessary.

## Dodatak

### Acknowledgements

Not applicable.

### Funding 

Not applicable.

### Availability of data and materials

The datasets used and/or analysed during the present study are available from the corresponding author upon reasonable request.

### Conflict of interest statement

All the authors declare that they have no conflict of interest in this work.
